# Durotaxis and extracellular matrix degradation promote the clustering of cancer cells

**DOI:** 10.1016/j.isci.2025.111883

**Published:** 2025-01-24

**Authors:** Mykhailo Potomkin, Oleg Kim, Yuliya Klymenko, Mark Alber, Igor S. Aranson

**Affiliations:** 1 Department of Mathematics, University of California, Riverside, Riverside, CA 92521, USA; 2 Interdisciplinary Center for Quantitative Modeling in Biology, University of California, Riverside, Riverside, CA, USA; 3 Department of Biomedical Engineering and Mechanics, Fralin Biomedical Research Institute, Center for Soft Matter and Biological Physics, Virginia Tech, Blacksburg, VA 24061, USA; 4 Department of Cell and Developmental Biology, Perelman School of Medicine, University of Pennsylvania, Philadelphia, PA 19104, USA; 5 Department of Obstetrics and Gynecology, Indiana University School of Medicine, Indianapolis, IN 46202, USA; 6 Mathematical Institute, Leiden University, Leiden, the Netherlands; 7 Department of Mathematics, Pennsylvania State University, University Park, PA 16802, USA; 8 Department of Biomedical Engineering, Pennsylvania State University, University Park, PA 16802, USA; 9 Department of Chemistry, Pennsylvania State University, University Park, PA 16802, USA

**Keywords:** biocomputational method, cancer systems biology, *in silico* biology

## Abstract

Early stages of metastasis depend on the collective behavior of cancer cells and their interaction with the extracellular matrix (ECM). Cancer cell clusters are known to exhibit higher metastatic potential than single cells. To explore clustering dynamics, we developed a calibrated computational model describing how motile cancer cells biochemically and biomechanically interact with the ECM during the initial invasion phase, including ECM degradation and mechanical remodeling. The model reveals that cluster formation time, size, and shape are influenced by ECM degradation rates and cellular compliance to external stresses (durotaxis). The results align with experimental observations, demonstrating distinct cell trajectories and cluster morphologies shaped by biomechanical parameters. The simulations provide valuable insights into cancer invasion dynamics and may suggest potential therapeutic strategies targeting early-stage invasive cells.

## Highlights

• Computational model reveals clustering of ovarian cancer cells in early invasion
• Model links cell dynamics with ECM degradation and mechanical interactions
• Durotaxis drives clustering and elongation of ovarian cancer cell aggregates
• MMP-induced substrate degradation affects cluster cell displacement and dynamics

## Graphical abstract

**Figure:**
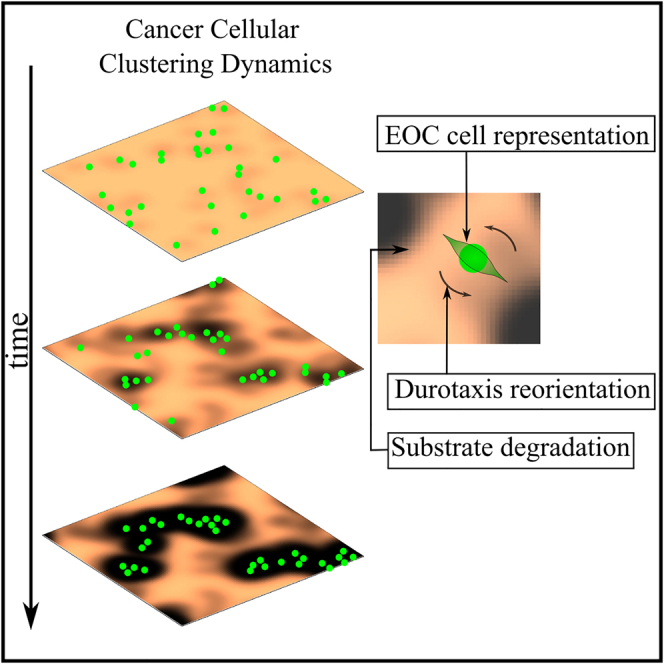

## Introduction

The characteristic feature of metastasis is the spread of cancer cells detached from primary tumors to distant areas of the body via the lymph system, bloodstream, or peritoneal fluid, the latter being common for epithelial ovarian cancers (EOCs).^1^^,^^2^^,^^3^ EOC-metastasizing cells initially attach to a single layer of mesothelial cells of the peritoneal lining inducing their retraction and exposure of the underlying extracellular matrix (ECM).^4^ The latter permits EOC cells to anchor to the underlying type I collagen-rich submesothelial matrix, followed by invasion into the matrix and subsequent formation of a widely disseminated metastatic lesion and tumor growth.^5^^,^^6^ Adhered to the submesothelial matrix, EOC multicellular aggregates (MCAs) are the key unit of metastatic dissemination.^7^^,^^8^ Previous studies reveal that EOC cancer cells tend to form aggregates at the absence of matrix adhesion with the number and size of the aggregates correlating with survival.^9^ Additionally, cells comprising MCAs were shown to be more resistant to anoikis, apoptotic cell death in response to loss of ECM attachment, than single cells. Cell aggregation also correlated with tumorigenicity *in vivo*.^10^ Despite its functional importance for EOC metastasis, the mechanisms of the MCA formation remain unclear. In particular, it is unknown how the physical and biochemical interactions between EOC cells and the submesothelial collagen-rich matrix can affect the formation of EOC clusters.

We developed a computational model that considers EOC cells crawling on the submesothelial ECM substrate of the peritoneum lining. The model is cell based,^11^ that is, each EOC cell is determined by its center of mass, motility, and orientation. Besides having the ability to crawl, the EOC cells can attach to and degrade the substrate, which in turn reduces the ability of EOC cells to move along the substrate. Cell-induced degradation of the ECM leads to the formation of voids within the matrix, assisting invasion. If a region of the substrate is fully degraded, a cell cannot move along this region. In addition, cells exert mechanical stress leading to in-plane displacements of the substrate. Each cell “senses” the overall stress and reorients accordingly, a phenomenon referred to as *durotaxis*.^12^^,^^13^

Several computational models of collagen substrate rearranged by cells were developed in the context of wound healing.^14^^,^^15^ In these models, cells (such as fibroblasts) migrate on an anisotropic layer with their movement governed by the geometric arrangement of collagen bundles. A combination of experiments and computational approach was used in the study by Hagiwara et al.^16^ to investigate how active remodeling and degradation of the ECM can facilitate the directionality of cell migration. Studies^17^^,^^18^^,^^19^ demonstrated that cells can degrade chemoattractants, thereby creating a self-generated chemical gradient and enhancing chemotaxis—the movement of cells toward a chemical concentration gradient. Other mechanisms for modifying the environment, such as the secretion and deposition of biochemical cues by cells to provide spatial memory and guidance for other cells, have also been modeled.^20^^,^^21^

In our model of EOC cell movement, the reorientation of cells is governed by durotaxis,^22^ that is, a cell tends to orient toward a stiffer region of the substrate. There is a variety of models of durotaxis in which cell movement is described using (1) Langevin dynamics with a noise term whose probability distribution function is biased to a stiffer region,^23^ (2) a random walk with a longer time of moving in the same direction (i.e., persistence time) on a stiffer region,^24^ (3) orientation dynamics depending on the distribution of individual focal adhesion complexes,^25^^,^^26^ (4) a cellular Potts model,^27^^,^^28^^,^^29^^,^^30^ or (5) a combination of the continuous formulation for substrate deformation with the agent-based approach for the durotactic cells.^31^^,^^32^ The response of cancer cells to both chemical and mechanical cues was recently studied in the study by Esfahani et al.^33^ Meanwhile, the synergistic effect of substrate remodeling and durotaxis on cancer cell dynamics is mostly unknown.

In this study, we explore the collective motion of EOC cells, with their individual dynamics coupled via mechanical interactions of the cells with the ECM inducing horizontal displacement of the matrix in the presence of the cell-induced proteolytic degradation of the matrix. Our focus is on the initial stage of cancer cell invasion, during which cells crawl along the surface of the ECM substrate and degrade it prior to invasion. At this stage, all interactions between cells and their displacements are effectively confined to a horizontal plane, allowing us to reduce our model to two dimensions. We note that the model can be directly extended to three spatial dimensions, and with appropriate adjustments informed by experimental data, may also be adapted to simulate later stages of metastasis.

Our findings reveal that the collective behavior of the cells results in their clustering, with spatiotemporal dynamics being highly sensitive to durotactic parameters and substrate degradation rate. The study’s outcome is a biophysical minimal model of EOC cell dynamics on collagen substrate with defined key characteristic parameters that can potentially be used for examining the response of EOC to drug treatment. Comparison of collective cell behaviors in simulations and experiments can assist in the evaluation of parameters that are difficult or impossible to measure in experiments (like durotaxis parameters, chemical reaction rates, degradation rates, etc.; see Table 1).

**Table 1:** Physical parameters and their dimensional values or their ranges

Parameter	Value/range	Description and source of data
$N_{c}$	30, 40, and 100	the number of cells in 0.25 ${\text{mm}}^{2}$^34^
L	0.5 mm	box size^34^
d	10 μm	cell’s diameter^34^^,^^35^
$V_{\text{prop}}$	$2\;\mu m\;h^{-1}=5.5\times 10^{-10}\;m\;s^{-1}$	cell’s propulsion speed^34^
$\phi_{0}$	10 $m\;s^{-1}$	soft steric interaction strength^34^
$E_{0}$	3 kPa	the substrate’s Young modulus^22^
ϵ	0.5	elasticity variation (due to ρ) coefficient∗
$k_{\sigma }$	$5.5\times 10^{2}$$s^{-1}$	coefficient relating displacement to the moment of force exerted by the substrate on cell∗
η	1−10 Pa	substrate friction coefficient^36^
α	$10^{-3}$$N\;m^{3}$	resistance to in-plane deformations^36^
$D_{C}$	$1.2\times 10^{-12}\;m^{2}\;s^{-1}$	the chemical diffusion coefficient∗
$C_{0}$	$5\times 10^{-11}\;\text{mol}\;s^{-1}$	the chemical production rate∗
$\gamma_{1}$	$5.5\times 10^{-5}\;s^{-1}$	the decay rate of the chemical due to its use to degrade substrate∗
β	$2\times 10^{-3}\;s^{-1}$	the chemical decay rate due to other reasons∗
$\gamma_{2}$	$5.5\times 10^{-7}\;m^{2}\;s^{-1}$	the degradation rate of the substrate∗

Values of parameters are taken of the same order as in indicated sources but may not coincide exactly. We mark with ∗ those parameters whose values were hypothesized, and no reliable source was found.

The relevance of our results is two-fold. Firstly, we provide an insight into the biophysical mechanism of cancer cell clustering that is important for the early stages of ovarian cancer metastasis formation. Secondly, we introduce a new class of agent-based models for the description of the collective behavior of self-propelled interacting particles.^37^ The majority of active matter systems deal with interacting self-propelled particles with local velocity or direction alignment interactions (so-called “dry active matter”).^38^ Another example is active particles in the fluid environment, e.g., microswimmers.^39^ In this situation, dominating interactions among the agents are hydrodynamic, like in the case of swimming bacteria.^40^^,^^41^ In our case, the interactions between cells are governed by elastic forces with very different spatiotemporal responses than the hydrodynamic drag. Furthermore, substrate degradation by the cells introduces a long-term memory effect that is not present in standard active matter models. The substrate degradation results in a number of non-trivial effects, like the formation of a “wake” or “ant-trail” behind the cell and a tendency of a cell to follow the wake. A somewhat similar behavior was observed experimentally in a different system: swimming bacteria in liquid crystals with a homeotropic director alignment.^42^

Overall, our findings provide insight into how various physical factors (peritoneum ECM stiffness and elasticity), as well as cell motility, impact the initial stages of EOC metastases that can suggest potential strategies for EOC metastases prevention by targeting ECM stiffness, density, or the ECM degradation rate.

## Results

To study the effect of durotaxis and ECM degradation on cancer cell clustering, we developed a continuum computational model of cancer cell invasion into ECM. The model is based on a system of coupled partial differential equations describing the motion of $N_{c}$ cells interacting with each other and a substrate. The *i*th cell is characterized by its location $r_{i}\left(t\right)$ and orientation $\varphi_{i}\left(t\right)$, defined as the angle between the self-propulsion direction of the *i*th cell and *x* axis.

Cells are assumed to be self-propelled entities exhibiting autonomous persistent motion in the absence of interactions with other cells. Cell motility is the result of a complicated sequence of biochemical and bio-mechanical events including polymerization of actin at the membrane, activity of motor proteins such as myosin, and formation of adhesion connections with the substrate.^43^^,^^44^^,^^45^ All the forces actively pushing the membrane are balanced by friction with the substrate to maintain motility. The equation describing the force balance in the overdamped limit is a differential equation $-\eta \dot{r}+f_{\text{prop}}p\left(\varphi \right)=0$, that is, the effective friction $-\eta \dot{r}$ is balanced by the active force $f_{\text{prop}}p\left(\varphi \right)$. Here, *η* is the friction coefficient, $f_{\text{prop}}$ is the propulsion strength, and p(φ)=(cos(φ),sin(φ)) is the cell orientation vector. For the fixed *φ*, this equation describes the motion of the cell along vector p(φ) with speed $V_{\text{prop}}=f_{\text{prop}}/\eta$. In our model, the cell motion is also affected by substrate degradation reducing $f_{\text{prop}}$, interactions with other cells, and the orientation angle changes due to durotaxis and rotational diffusion (see Equations 1 and 2).

Epithelial ovarian cancer cells secrete matrix metalloproteinases (MMPs), enzymes whose activity leads to the degradation of the collagen substrate.^46^^,^^47^ In our model, the local concentration of cell-produced MMPs is described by the function C(x,t), and the substrate is degraded by MMPs in a concentration-dependent manner. To model substrate degradation, we introduce function ρ(x,t) such that 0≤ρ(x,t)≤1 and 1−ρ(x,t) can be viewed as the degree of ECM degradation around point x at time *t*. Namely, if ρ(x,t)≈1, then the substrate is close to its initial (undisturbed) state, and cells can freely move in the vicinity of the point x. At ρ(x,t)≈0, the substrate is fully degraded in the vicinity of the point x, and cells slow down or stop when they pass through the point x. Mathematically, the effect of substrate degradation on cell motility is described by the first term in the left-hand side of the Equation 1.

**Figure 1:**
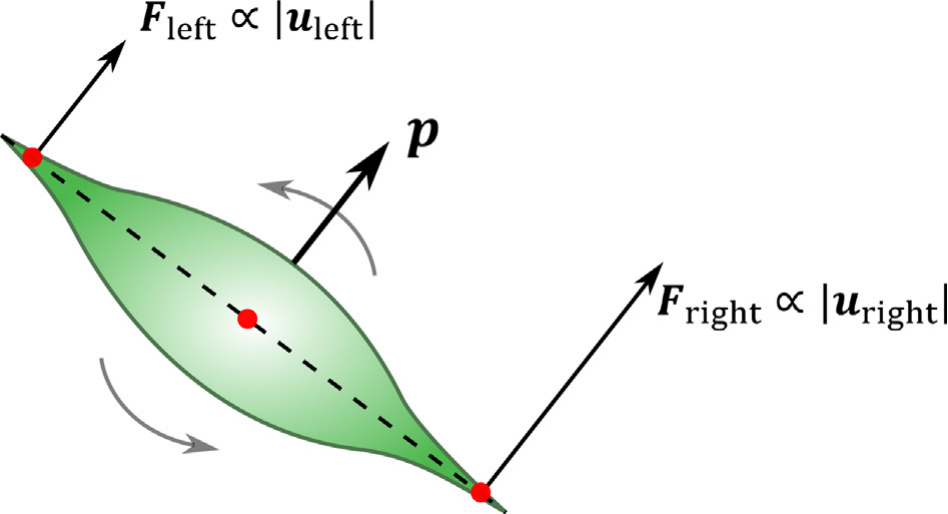
Schematics of cell durotaxis implementation Green domain represents an individual cell. Vector p=(cosϕ,sinϕ) shows the cell orientation. The reorientation of the cell due to durotaxis is described by competition of two pulling forces on the cell’s sides $F_{\text{right}}$ and $F_{\text{left}}$, which are proportional to the magnitude of local substrate displacements $u_{\text{right}}$ and $u_{\text{left}}$, respectively.

We model durotaxis as follows. A cell transfers its propulsion forces on the substrate through adhesive complexes to move persistently. The complexes form and eventually break, allowing the cell to displace over large distances. Rupture of an adhesive connection may occur if it becomes too extended (see, e.g., the study by Erdmann and Schwarz and Sabass and Schwarz^48^^,^^49^ for modeling such dynamics on the part of the membrane, and Ziebert and Aranson^50^ on the entire cell). That is the case when the endpoint of an adhesive connection on the deformed substrate is significantly displaced. To take this into account, we assume that the adhesivity of the substrate beneath the cell decreases with the magnitude of substrate displacement u. For example, let the right tip of the cell be located above a more deformed substrate than the left one. Then, the right tip becomes less adhesive, forcing the cell to reorient counterclockwise. This reorientation is visualized in Figure 1 as the torque resulting from the application of the force couple, $F_{\text{left}}$ and $F_{\text{right}}$, involved in self-propulsion, thus parallel to the orientation vector p, and both forces are proportional to |u|. We note that our simplifying assumption—that cell adhesion to the substrate is higher at contacts with greater substrate displacement—allows us to capture durotactic behavior in a computationally feasible manner. The actual process of formation and breakage of adhesive connections, especially, with a deformable substrate, is highly complex and stochastic. Other approaches describing orientation-dependent cellular responses to mechanical cues have been developed.^23^^,^^24^^,^^25^^,^^28^^,^^29^^,^^51^ Additionally, the model does not account for cellular reorientation toward stiffness gradients, as it remains unclear whether cells sense stiffness gradients or instead respond to substrate strain,^52^ or stress.

By moving on the elastic substrate, all cells exert the cell traction force and thus generate in-plane stress within the substrate. Reorientation of the cell from the softer region of the substrate toward the stiffer one is implemented by computing the substrate displacement, u, around the cell (Figure 1). When displacement u on a side of the cell is larger than the one on the other side of the cell, the cell reorients toward a smaller displacement; see the first term in the left-hand side of Equation 2.

The governing equations of the model are written for unknown cells’ locations and orientations ${\left\{\left(r_{i},\varphi_{i}\right)\right\}}_{i=1,N_{c}}$, horizontal displacement of collagen substrate u(x,t), MMP concentration C(x,t), and the substrate parameter ρ(x,t):

(Equation 1) $$\partial_{t}r_{i}=V_{\text{prop}}\rho p_{i}+\sum_{j=1,j\neq i}^{N_{c}}\varphi \left(\left|r_{j}-r_{i}\right|\right)\frac{r_{j}-r_{i}}{\left|r_{j}-r_{i}\right|},i=1,..,N_{c}\text{,}$$

(Equation 2) $$\partial_{t}\varphi_{i}=k_{\sigma }\left(\left|u\left(x_{\text{right}},t\right)\right|-\left|u\left(x_{\text{left}},t\right)\right|\right)+\sqrt{2D_{\text{rot}}}\dot{\zeta }\text{,}$$

(Equation 3) $$\eta \partial_{t}u=\nabla \cdot \sigma +T,\sigma =\left[E_{0}\left(1+\epsilon \rho \right)/2\right]\left(\nabla u+{\left(\nabla u\right)}^{T}\right)\text{,}$$

(Equation 4) $$\partial_{t}C=D_{C}\Delta C+\sum_{i=1}^{N_{c}}w\left(\left|x-r_{i}\right|\right)-\gamma_{1}C\rho -\beta C\text{,}$$

(Equation 5) $$\partial_{t}\rho =-\gamma_{2}C\rho \text{.}$$

Equation 1 describes the dynamics of the cell location. The first term in the right-hand side of the Equation 1 accounts for the propulsion speed $V_{\text{prop}}$. Since the actual dependence of propulsion speed on *ρ* for 0<ρ<1 is not accessible, we assume the simplest linear dependence on *ρ*. The propulsion speed is maximal and equals to $V_{\text{prop}}$ if the substrate is not disturbed, ρ=1, whereas cells cannot propel themselves on the fully degraded substrate, ρ=0. Our modeling assumption is that cell velocity is influenced by substrate degradation, while the primary effect of substrate deformations on cell’s dynamics is cell reorientation, the first term in Equation 2. The second term in the right-hand side of Equation 1 accounts for cell-cell steric repulsion at a small distance to prevent an overlap of cells. These steric interactions are modeled by φ=∇Π(|x|), where $\Pi \left(r\right)=F_{0}e^{-r^{2}/l_{0}^{2}}$. Equation 2 describes cell re-orientation due to durotaxis and rotational diffusion by the first and second term in the right-hand side in Equation 2, respectively. Here, $k_{\sigma }$ is the durotaxis parameter, $D_{\text{rot}}$ is the rotation diffusion coefficient, and $\dot{\zeta }$ is an uncorrelated noise with the intensity $\left\langle \dot{\zeta }\left(t^{\prime }\right)\dot{\zeta }\left(t^{\prime \prime }\right)\right\rangle =\delta \left(t^{\prime }-t^{\prime \prime }\right)$. Equation 3 describes dynamics of the horizontal displacement u, which is determined by friction damping $\eta \partial_{t}u$, elastic stress *σ*, and traction force exerted by cells $T\left(x\right)=\sum_{i=1}^{N_{c}}T_{i}\left(x\right)$^36^^,^^53^. The force field $T_{i}$ exerted by the $i^{\text{th}}$ cell on the substrate is localized within the circular domain $\left|x-r_{i}\right|<d/2$. For the purpose of computing the traction force, we represent the cell shape as a disc with a diameter *d*. Additionally, similar to the approach in the study by Ziebert and Aranson,^50^ we assume that each cell exerts the zero net force on the substrate: $\int T_{i}\;dr=0$ for $i=1,..,N_{c}$. We assume that the traction force for each cell is parallel to its polarization, that is, $T_{i}\left(x\right)={\hat{T}}_{i}\left(x\right)p\left(\varphi_{i}\right)$ where ${\hat{T}}_{i}$ is a localized scalar function having zero average; the specific form of ${\hat{T}}_{i}$ used in simulations can be found in Figure S3. Note that the elastic coefficient $E_{0}\left(1+\epsilon \rho \right)$ decreases as *ρ* decreases: when substrate degrades, it gets softer. The linear dependence of the elastic coefficient on *ρ* is consistent with our experimental observation of mechanical and structural properties of a collagen matrix, see Figure S1. The rate of change of the chemical *C* is the combination of chemical diffusion $D_{C}\Delta C$, rate of the chemical production by cells $\sum_{i=1}^{N_{c}}w\left(\left|x-r_{i}\right|\right)$ (here $w=\nu_{C}\;\exp\;\left\{-{\left|x-r_{i}\right|}^{2}/d^{2}\right\}$ is the “weight” of a cell at a given location), removal of the chemical to degrade the substrate $-\gamma_{1}C\rho$, and degradation of the chemical by itself, −βC; see all the terms in the right-hand side of Equation 4. Finally, the local degradation of the collagen substrate in terms of the parameter *ρ* by the chemical *C* is described by Equation 5. Description and specific values of problem parameters $V_{\text{prop}}$, $k_{\sigma }$, $D_{\text{rot}}$, *η*, $E_{0}$, *ϵ*, $D_{C}$, $\gamma_{1}$, *β*, $\gamma_{2}$, and *d* are provided in Table 1.

### Model simulations

#### Clustering of cells in the presence of substrate degradation and mechanical interaction with the ECM

**Figure 2:**
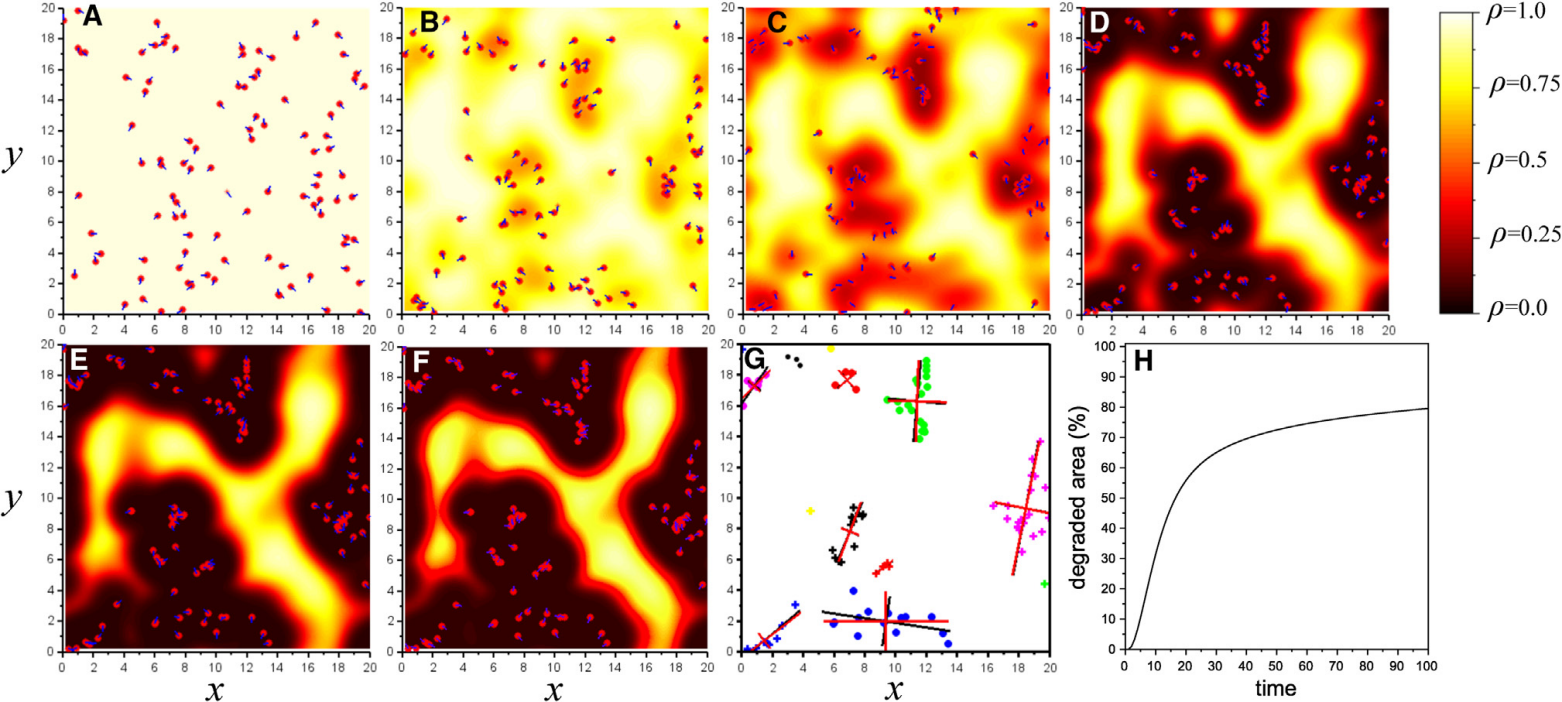
Spatiotemporal dynamics of individual cancer cells, in the presence of cell-mediated substrate degradation and mechanical interactions (A–F) Representative snapshots of simulated cells and the extent of substrate degradation in a computational domain of size $L^{*}\times L^{*}$ shown at t=0,5τ,10τ,30τ,60τ,80τ (*τ* = 4 h). The non-dimensional domain size is $L^{*}=L/l$, with *L* = 0.5 mm and *l* = 25 μm. Cells are depicted as small red circles with blue segments indicating their propulsion direction. Substrate degradation characterized by the condition order parameter, *ρ*, is visualized as a color map. See Video S1 for a full sequence of the simulation. (G) Spatial arrangement of cell clusters and cell distribution within clusters at $T_{\text{degr}}$, when cell movement ceases. Cells are considered part of the same cluster if the distance between them is less than 50 μm (2 units of length) and are shown with the same color and marker (a small cross or circle). Large crosses indicate the major and minor axes of clusters, with the larger segment representing the major axis. Cluster axes were calculated using geometric (black lines) and covariance matrix (red lines) methods (see text). (H) Area of the substrate degraded by cells as a function of non-dimensional time, T/τ.

To study the dynamics of migrating cells producing ECM-degrading MMPs and exhibiting durotaxis, we randomly distributed $N_{c}$ cells with diameter d=10μm on the surface of a L×L substrate with L=0.5mm and periodic boundary conditions, see Figure 2A. Cells’ orientations at t=0 are random as well. The substrate is initially unperturbed, i.e., ρ≡1. Cells crawl freely on the substrate ρ≡1 with propulsion speed $V_{\text{prop}}=2\mu m\;h^{-1}$.

Video S1. Cell dynamics and substrate degradation

Our simulations showed that upon migration, cells form different size clusters (Figure 2), which are defined as a group of cells such that each cell in the group has a neighbor from this group at the maximum distance of 50 μm. To assess cell cluster dynamics, we computed two differently-averaged cluster numbers $S_{1}$ and $S_{2}$ ^54^^,^^55^:

(Equation 6) $$S_{1}=\frac{q_{1}}{q_{0}}\;\text{and}\;S_{2}=\frac{q_{2}}{q_{1}},\text{where}\;q_{k}=\sum_{m=1}^{\infty }m^{k}n_{m}\text{.}$$

**Figure 3:**
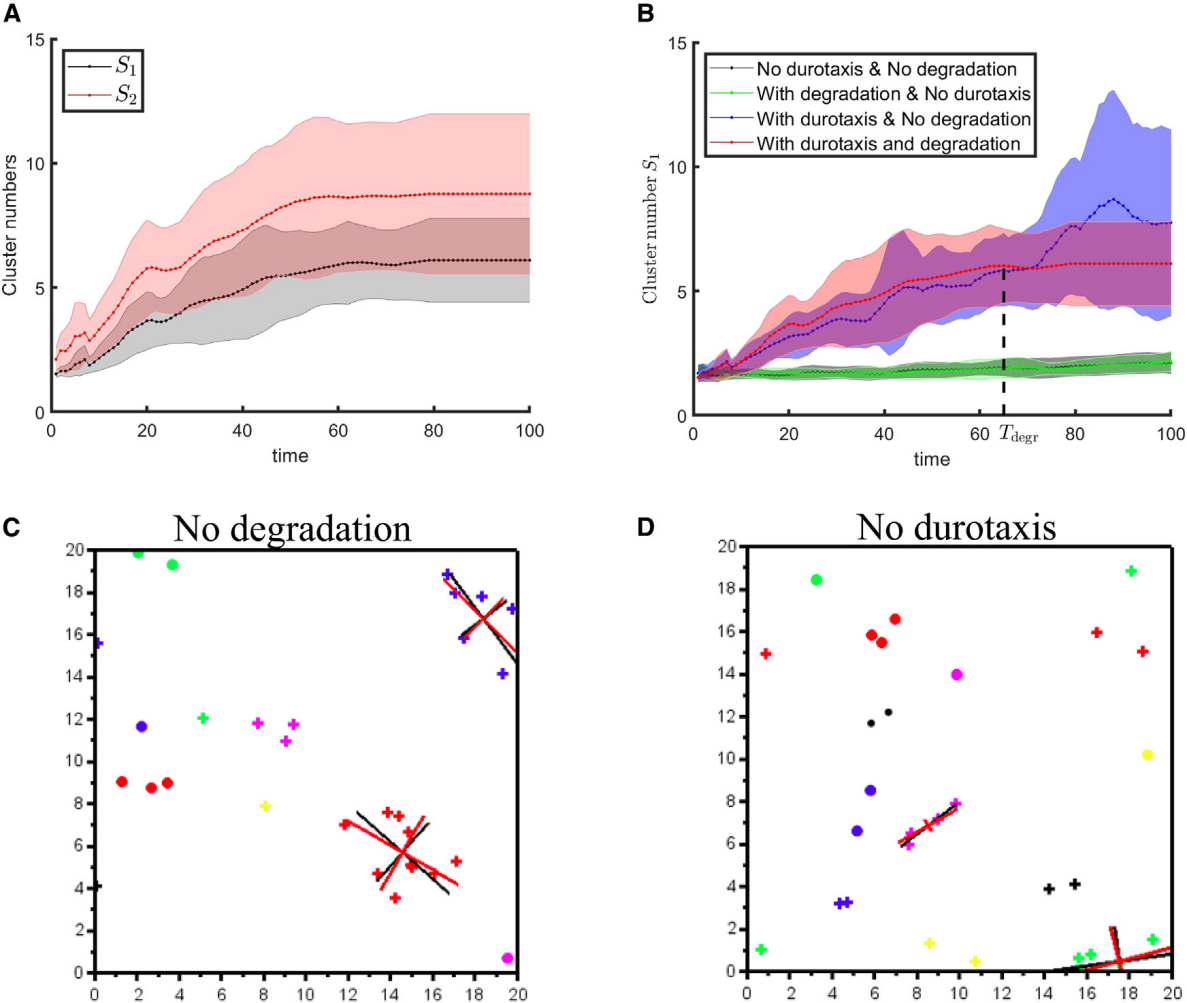
Effects of durotaxis and matrix degradation on cell clustering dynamics (A) Temporal changes in cell cluster numbers ($S_{1}$ and $S_{2}$), as defined by Equation 6. (B) Temporal dynamics of cluster size $S_{1}$ under four conditions: with/without either durotaxis or substrate degradation. $T_{\text{degr}}$ marks the average time when cells become immobilized due to complete substrate degradation (ρ≈0) near cells. Solid lines and shaded areas in (A) and (B) represent the mean and standard deviation based on n=20 simulations with $N_{c}=40$ cells, that is, data are represented as mean ±$\sqrt{n}\text{SEM}$. The horizontal *x* axis represents a dimensionless time T=t/τ, with *τ* = 4 h. (C and D) Representative numerical simulations of cell clustering in the absence of matrix degradation (C) or durotaxis (D) at *T* = $T_{\text{degr}}$, illustrating that matrix degradation results in more elongated cell clusters. Simulation parameters match those in Figure 2G. Additional examples of cell cluster dynamics under different durotaxis and substrate degradation conditions are provided in Videos S2, S3, S4, and S5.

Here, $n_{m}$ is the number of clusters containing *m* cells. Note that $q_{1}=\sum_{m=1}^{\infty }mn_{m}=N_{c}$ is the total number of cells and $q_{0}=\sum_{m=1}^{\infty }n_{m}$ is the total number of clusters. $S_{1}$ is the average number of cells in a randomly chosen cluster, and $S_{2}$ is the average number of cells in the cluster of a randomly chosen cell. Indeed, for a given set of cell locations at a fixed moment in time, the number $p_{m}=\frac{n_{m}}{q_{0}}$ represents the probability of selecting a cluster with *m* cells from all clusters. Then, $S_{1}={\left\langle m\right\rangle }_{p}=\sum_{m}mp_{m}$. Similarly, the number ${\hat{p}}_{m}=\frac{mn_{m}}{q_{1}}$ represents the probability of selecting a cell belonging to a cluster with *m* cells. Consequently, $S_{2}={\left\langle m\right\rangle }_{\hat{p}}=\sum_{m}m{\hat{p}}_{m}$. From the standard Cauchy-Schwarz inequality, it follows that $q_{1}^{2}\leq q_{0}q_{2}$, and thus $S_{1}\leq S_{2}$. The equality $S_{1}=S_{2}$ is attained if and only if at most one $n_{m}$ is non-zero, meaning all clusters are of the same size. For example, $S_{1}=S_{2}$ holds either in the case of a single large cluster or in the case of many small clusters of identical size. However, $S_{2}$ can be significantly larger than $S_{1}$, when a large cluster co-exists with multiple small clusters. Numerical values for $S_{1}$ and $S_{2}$ are depicted in Figure 3A: both numbers $S_{1}$ and $S_{2}$ grow with time up to $T_{\text{degr}}$ and remain constant at $t>T_{\text{degr}}$. The difference between $S_{1}$ and $S_{2}$ remains finite for all times, thus illustrating the formation of multiple clusters of various sizes observed in numerical simulations. The growth of cluster numbers $S_{1}$ and $S_{2}$ is non-monotonic due to the relatively small number of cells and the fact that cells constantly leave and enter clusters due to self-propulsion, which leads to fluctuations of cluster numbers. The growth of clusters stops at $T_{\text{degr}}$. Namely, simulations show that cell movement over the substrate by self-propulsion was halted at time $T_{\text{degr}}$, as a result of a significant increase in the extent of substrate degradation, ρ→0, in the vicinity of cell clusters.

Video S2. Cell dynamics and cluster numbers with both durotaxis and degradation

Video S3. Cell dynamics and cluster numbers with durotaxis and without degradation

Video S4. Cell dynamics and cluster numbers without durotaxis and with degradation

Video S5. Cell dynamics and cluster numbers without both durotaxis and degradation

To characterize the shape of cell clusters, we computed the major and minor axes (or, equivalently, orientation) in two complementary ways: by finding the axis minimizing the total distance to cells of the cluster and by finding the eigen-directions of the cluster covariance matrix, see STAR Methods. Two different methods for finding axes are used for cross-validation. As depicted in Figure 2E, the two ways of determination of the major axis lead to almost the same result. Next, clusters’ lengths are larger than their widths, illustrating their elongation. To explain elongation, note that self-propelled cells create degraded regions behind themselves. These regions become a trap for other cells that have to slow down or stop at these regions, further degrading the substrate. That may be reminiscent of the leader-follower behavior of epithelial cells^34^^,^^56^ in the sense that the dynamics of some cells determine the eventual location of other cells.

Production of MMPs by migrating cells resulted in the gradual degradation of the substrate around cells, as seen from the dark-color gradient areas in Figures 2B–2F. The size of ECM-degraded areas $\left(1-\frac{1}{L^{2}}\int_{{\left[0,L\right]}^{2}}\rho \left(x\right)\;dx\right)\times 100\%$ was found in simulations to be ≈70% at $t=T_{\text{degr}}$, see Figure 2H.

#### Durotaxis enhances clustering while degradation makes the clusters stable

To illustrate the role of both durotaxis and substrate degradation, we performed numerical simulations in four cases: (1) with both durotaxis and degradation, (2) without durotaxis and with degradation, (3) with durotaxis and without degradation, and (4) without durotaxis and degradation. Results for the average cluster size $S_{1}$ are depicted in Figure 3B. If cells do not interact elastically (no durotaxis), then large clusters (of three cells or more) do not typically form (see green and black curves in Figure 3B, which oscillate between $S_{1}$ = 1 and $S_{1}$ = 2). If cells exhibit durotaxis but do not degrade the substrate, then clusters do form, and their size may even exceed that in the case of both durotaxis and degradation. However, these clusters are unstable, they dissolve quickly after formation, and cells often change the clusters to which they belong. To further elucidate the impact of substrate degradation on the dynamics of durotactic cells, we computed the aspect ratios of cell clusters and the average cell speed at the final simulation step (*T* = 50). Results in Table 2 show that in the presence of substrate degradation, clusters are more elongated and static.

**Table 2:** Mean values and confidence intervals for the aspect ratio of cell clusters and cell speed (p=0.05)

	Durotaxis	Durotaxis and degradation
Cluster aspect ratio	1.7695±0.6607	2.2658±0.7152
Cluster cell speed	0.1768±0.00078	$6.1\times 10^{-4}\pm 2.4\times 10^{-5}$

The results are based on 40 simulations, each involving 20 cells. The total number of clusters is 64 for simulations without substrate degradation and 82 for those with degradation. The cell speed is non-dimensionalized as $v/v_{0}$, where $v_{0}=2\;\mu m\;h^{-1}$.

**Figure 4:**
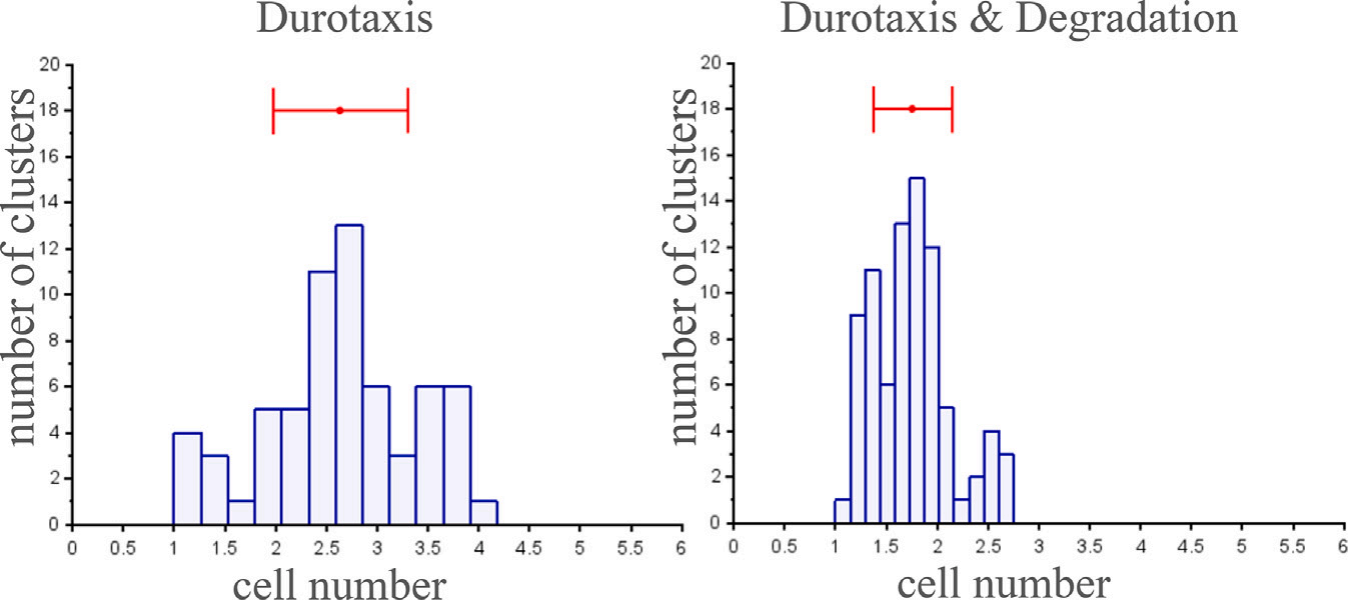
Connectivity index distributions for simulated cellular clusters in the absence and presence of substrate degradation The connectivity index for a given cluster is defined as the average number of cell neighbors in the cluster. The connectivity index is computed at the final time step of simulations (*T* = 50) for each cluster containing at least 4 cells. Results are based on 40 simulations each involving $N_{c}=20$ cells. The total number of clusters is 64 for simulations without substrate degradation and 82 for those with degradation. The red horizontal segment indicates the 95% confidence interval.

Additionally, we compare cluster connectivity indexes for cases with and without substrate degradation (Figure 4). The connectivity index for a given cluster is defined as the average number of cell neighbors in the cluster. Recall that two cells in the cluster are called neighbors if the distance between their centers is less than 50 μm. It is evident from Figure 4 that values of connectivity indexes are smaller for the case with degradation. A smaller connectivity index means that cells have fewer neighbors and the cluster is more elongated. Results in Figure 4 are another evidence that degradation makes cellular clusters more elongated.

Finally, we performed a sensitivity analysis for cluster size and connectivity in response to changes in degradation and durotactic parameters, $\gamma_{2}$ and $k_{\sigma }$. This was achieved by running multiple simulations, varying $\gamma_{2}$ and $k_{\sigma }$ over an order of magnitude range, while keeping other parameters constant at their typical values. The results shown in Figure S2 indicate that cluster sizes and their connectivity on a degraded surface (i.e., at $t>T_{\text{degr}}$) decrease 5-fold and 2-fold, respectively, as the degradation rate $\gamma_{2}$ increases 10-fold. This observation can be explained by the reduced time available for large clusters to form or elongate with higher degradation rates. In contrast, cluster numbers and connectivity indexes remain unchanged as durotaxis reorientation rate parameter $k_{\sigma }$ is increased 10-fold beyond its typical value. Thus, cluster size and connectivity were more sensitive to changes in degradation than in durotaxis.

### Experiment

#### Extracellular collagen-mediated clustering of epithelial ovarian cancer cells

**Figure 5:**
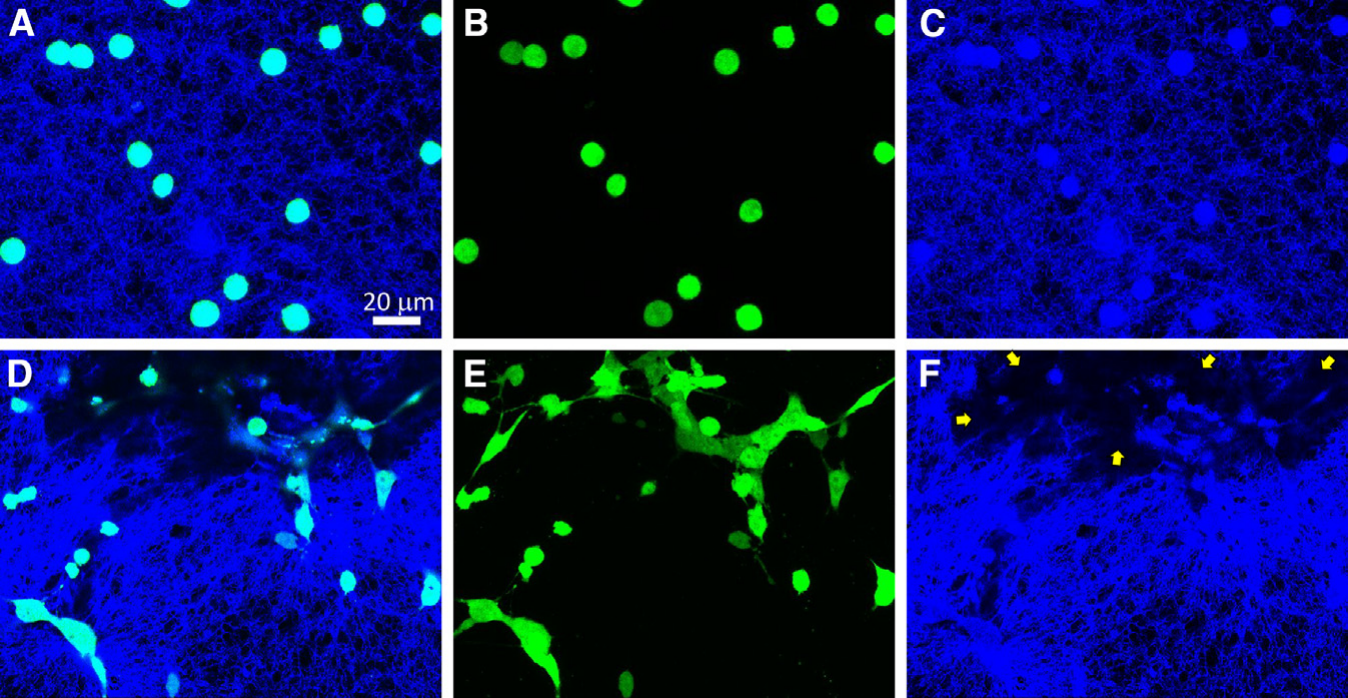
Collagen-mediated clustering of ovarian cancer cells and cell-driven matrix remodeling (A–C) Confocal microscopy images of individual DOV13 cells and collagen matrix immediately after application of the cells on top of the matrix (t=0). (D–F) Cell clustering accompanied by collagen degradation observed at 24 h of incubation, shown in a single confocal microscopy slice in (D) and (F) and in a *z*-projection of a portion of confocal microscopy z stack (E). The GFP-labeled DOV13 cells appear in green. Second-harmonic generation (SHG) microscopy reveals collagen and cell spatial relationship to the collagen in blue. The overlap of GFP fluorescence into the SHG channel is due to spectral bleedthrough as a broad SHG spectral range was used to optimize signal capture from collagen. Yellow arrows indicate areas of collagen degradation in (F).

To study if epithelial ovarian cancer cells form clusters in collagen upon initial invasion, DOV13 cells expressing green fluorescence protein (GFP) were seeded as individual cells on top of a collagen matrix. The cells were observed using a laser scanning microscope immediately after seeding and at 24 h of incubation. Confocal microscopy images of DOV13 cells reveal that at t=0, cells were uniformly dispersed over the collagen. In contrast, at t=24 h, single cells self-rearranged into a physically communicating cell-cell network on top of the collagen layer with some cells assembling into distinguishable clusters. Analysis of cell sizes indicated that cell clustering caused a significant 50% increase in the mean area of cellular objects, which were primarily composed of cell aggregates, measured in *z*-projections of confocal z stacks. Visualization of the collagen matrix using the reflectance mode of the microscope revealed that cell clustering was accompanied by collagen degradation distinct at 24 h (Figure 5). The extent of collagen degradation, measured as a relative collagen-free area of the matrix around cells, significantly increased by 32% at t=24 h compared to unperturbed collagen at t=0. Thus, the initial invasion of single DOV13 cells was accompanied by cell clustering and remodeling/degradation of the extracellular collagen matrix.

**Figure 6:**
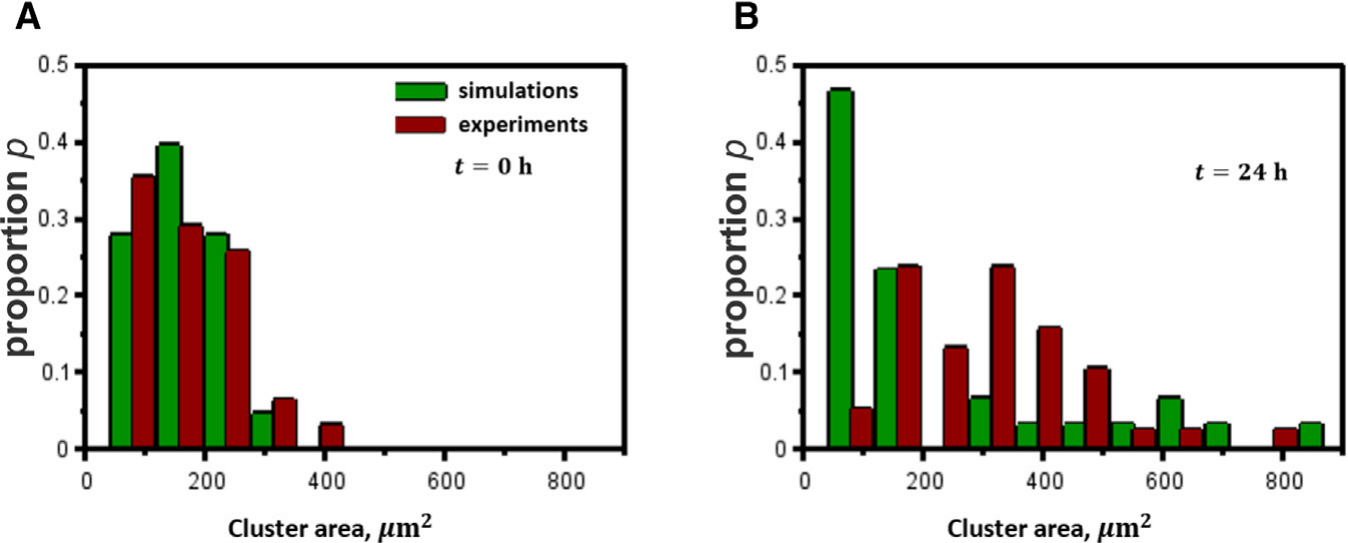
Comparative analysis of cell clustering in the collagen matrix assessed experimentally and using model simulations Cell cluster size distributions were assessed at 0 at 24 h of incubation and calculated as the ratio of the number of individual clusters within the respective area range to the total number of clusters. The horizontal axis represents the area of individual clusters, measured in $\mu m^{2}$. The proportion *p* is defined as the ratio of the number of observed clusters with an area within the given range to the total number of observed clusters. The bin width corresponds to the area of a single circular cell of diameter d=10μm. (A) depicts the initial cluster size distribution, whereas (B) depicts the cluster size distribution after 24 h.

The comparison of clustering between simulations and experiments by assessing the individual cell cluster area at t=0h and t=24h revealed qualitatively similar cell cluster size distributions (Figure 6). While the model assumes cells maintain their area, experimental observations show that cells tend to spread slightly, resulting in a broader peak of the cluster area distribution compared to simulations. Provided that more experimental data are available, the model can be calibrated to capture the cluster distributions more accurately.

## Discussion

The interaction between ovarian cancer cells and the peritoneum is a crucial aspect of ovarian cancer progression and metastasis. In this study, we employed a computational model to investigate the behavior of ovarian cancer cells on the substrate mimicking the lining of the peritoneal cavity. Our findings shed light on several important aspects of ovarian cancer cell behavior and its interaction with the ECM, providing valuable insight into underlying biophysical mechanisms of cellular behavior following their adhesion to the ECM.

Firstly, our results demonstrate that ovarian cancer cells exhibit collective behavior, wherein groups of cells interact and move in a coordinated manner. This collective behavior has been observed in various cancer types and plays a critical role in cancer progression and metastasis.^57^^,^^58^ Our computational results align with our experimental data revealing ovarian cancer cell clustering in the collagenous ECM as a result of collective behavior and with previous experimental studies,^59^^,^^60^ further supporting the validity and relevance of our model. Secondly, we demonstrate that the biomechanical interaction between cancer cells and the substrate leads to the formation of elongated shape clusters, where cancer cells organize themselves into elongated structures, being consistent with ovarian cancer cell cluster formation in the collagen matrix revealed in experiments. We also showed that the formation of elongated clusters of self-propelled cells cannot be obtained if either durotaxis or substrate degradation is negligible. While durotaxis alone leads to cell clustering, the elongation makes these clusters long lasting and elongated, leading the way to creation of network structures observed in experiments for ovarian cancer cells.^34^

Furthermore, we highlighted the role of durotaxis in enhancing cancer cell clustering. Our model demonstrates that the responsiveness of ovarian cancer cells to external mechanical stimuli promotes their clustering. This finding is supported by previous studies indicating the involvement of durotaxis in cancer cell migration.^61^ The identification of durotaxis as a contributing factor to cancer cell clustering further emphasizes the importance of considering the biomechanical properties of the substrate in understanding ovarian cancer behavior and invasion.

In addition, our computational model incorporates the degradation of the substrate by MMPs. MMPs are enzymes involved in ECM remodeling and ovarian cancer progression.^62^ In ovarian cancer, MMPs were shown to regulate multicellular aggregate formation.^63^ Our simulations show that substrate degradation by MMPs decreases the transversal displacement of cancer cells within the clusters, therefore impacting their size distribution. The proteolytic degradation of the ECM by MMPs limits cell clustering following anchoring, while promoting cancer cell invasion revealed previously.^64^

The implications of our results extend beyond getting better understanding of ovarian cancer cell behavior. The identification of collective behavior, the role of biomechanical interactions, and the influence of durotaxis and substrate degradation by MMPs provide valuable information for the development of potential therapeutic strategies. Targeting the mechanisms underlying cancer cell clustering, such as modulating durotaxis or inhibiting MMP activity, may offer new avenues for intervention and prevention of metastasis.

Our model is two-dimensional and is designed to study the initial stage of cancer invasion, where EOC cells crawl on the substrate. The model can be extended to three dimensions by incorporating the spatial variable *z*. The primary modification involves replacing Equation 2, which governs the azimuthal angle *φ*, with an equation for the unit orientation vector $p\left(\varphi ,\theta \right)={\left(\cos\left(\varphi \right)\sin\left(\theta \right),\sin\left(\varphi \right)\sin\left(\theta \right),\cos\left(\theta \right)\right)}^{T}$ of the cell’s polarization direction, where *θ* is the inclination angle. Specifically, this equation can be written in the general form $\dot{p}=\omega \times p+\sqrt{2D_{\text{rot}}}\dot{\zeta }$. In the two-dimensional case, Equation 2 can be recovered by defining ω as $\omega =k_{\sigma }\left(\left|u\left(x_{\text{right}}\right)\right|-\left|u\left(x_{\text{left}}\right)\right|\right)e_{z}$, with $e_{z}={\left(0,0,1\right)}^{T}$. Testing various expressions for ω and comparing with experimental data will help elucidate general principles of cancer cell orientation dynamics during invasion. In the current two-dimensional model, the propulsion velocity is proportional to the substrate parameter *ρ*, as the model considers only horizontal cell displacements. This results in zero propulsion velocity when ρ≈0 around the cell, indicating complete substrate degradation and vertical cell invasion. In a three-dimensional extension, which accounts for both horizontal and vertical components of motion, it would be reasonable to remove the dependence of propulsion speed on *ρ*. Instead, a reaction force exerted by the degraded substrate could be introduced. Finally, at later stages of invasion, cells are known to move collectively. Collective durotaxis was shown to be more efficient than the durotaxis of isolated cells.^32^^,^^65^ The roles of adhesion complexes and their types are crucial for collective durotaxis.^32^^,^^34^ Incorporating cell-cell adhesion interactions into the three-dimensional model will therefore be essential for accurately describing the dynamics of invading clusters.

### Limitations of the study

While durotaxis and degradation are key processes influencing the dynamics of individual ovarian cancer cells and their clustering, as well as the behavior of attached MCAs, these processes are accompanied by other factors, such as cell proliferation, cell-cell adhesion, and shape dynamics (e.g., polarization). Among these, cell proliferation plays a particularly significant role in increasing the size of ovarian cancer MCAs during the later stages of invasion.

Isolating the effects of durotaxis and degradation is challenging due to their interdependence—matrix degradation modifies stiffness gradients, while durotaxis depends on ECM remodeling. Furthermore, these processes share overlapping molecular pathways and involve dynamic interactions, making it difficult to decouple them experimentally.

With additional experimental data, the model can be further refined to more accurately capture cluster distributions and dynamics in 3D.

## Resource availability

### Lead contact

Requests for further information and resources should be directed to and will be fulfilled by the lead contact, Mykhailo Potomkin (mykhailp@ucr.edu).

### Materials availability

This study did not generate new materials.

### Data and code availability

• Data: All data reported in this paper, derived from both simulations and experiment, can be shared by the lead contact upon request.
• Code: The code to carry out the simulations is publicly available at Mendeley https://data.mendeley.com/datasets/rb6zzz47fz/1.
• Any additional information required to reanalyze the data reported in this paper is available from the lead contact upon request.

## Acknowledgments

The work of Igor Aranson is supported by 10.13039/100000001NSF award PHY-2140010. M.A. was partially supported by 10.13039/100000001NSF grant MODULUS DMS-2029814 and NSF grant DMS 2424826. M.A. also gratefully acknowledges partial financial support for his research by the 10.13039/100022627Fulbright U.S. Scholar Program, which is sponsored by the U.S. Department of State, and the 10.13039/100021070Stichting Fulbright Commission the Netherlands. Its contents are solely the responsibility of the author and do not necessarily represent the official views of the Fulbright Program, the Government of the United States, or the Stichting Fulbright Commission the Netherlands.

## Author contributions

M.P., O.K., M.A., and I.S.A. developed the model, analyzed the results of numerical simulations, and wrote the paper. O.K. and Y.K. carried out the experiments.

## Declaration of interests

The authors declare no competing interests.

## STAR★Methods

### Key resources table

**Table:** 

REAGENT or RESOURCE	SOURCE	IDENTIFIER
**Chemicals, peptides, and recombinant proteins**		

Minimal Essential Medium (MEM)	Gibco	Cat# 11095080
Fetal Bovine Serum	Gibco	Cat# 16000044
MEM Non-Essential Amino Acids	Gibco	Cat# 11140-050
Penicillin-Streptomycin	Lonza	Cat# DE17-602E
Sodium Pyruvate	Corning Cellgro	Cat# 25000CI
Amphotericin B	Corning Cellgro	Cat# 30-003-CF
Insulin, Human Recombinant Zinc Solution	Gibco	Cat# 12585014
Rat Tail Collagen I	Corning	Cat# 354236
Phosphate Buffered Saline, pH 7.4	Gibco	Cat# 10010-023

**Experimental models: Cell lines**		

DOV13 Human Ovarian Carcinoma Cell Line	Dr. Stack Lab (Harper Cancer Research Center, South Bend, IN)	Cell line was authenticated by Genetica DNA Laboratories using short tandem repeat DNA profiling and was found to be >95% concordant.

**Software and algorithms**		

Fiji	https://imagej.net/software/fiji/	Ver.1.54f
GraphPad 10	GraphPad	https://www.graphpad.com/features
MATLAB analysis script	this paper: https://data.mendeley.com/datasets/rb6zzz47fz/1	https://doi.org/10.17632/rb6zzz47fz.1
C code	this paper https://data.mendeley.com/datasets/rb6zzz47fz/1	https://doi.org/10.17632/rb6zzz47fz.1

**Other**		

PELCO glass bottom dishes	*In Vitro* Scientific	#D35-20-4171-N

### Experimental model and study participant details

#### Cell line

The EOC DOV13 cell line was kindly provided by Dr. Robert Bast (M.D. Anderson Cancer Center, Houston, TX, USA), fluorescently tagged with GFP via lentiviral transduction, and maintained in Dr. Stack Lab (Harper Cancer Research Center, South Bend, IN) as follows.^34^ DOV13 cells were maintained in Minimal Essential Medium (Gibco, Big Cabin, OK, USA) containing 10% fetal bovine serum (Gibco), 1% Non-Essential Amino Acids (Corning Cellgro, Manassas, VA, USA), 1% Penicillin/Streptomycin (Lonza, Allendale, NJ, USA), 1% Sodium Pyruvate (Corning Cellgro) and 0.1% Amphotericin B (Cellgro) and supplemented with 10 *μ*g/ml Insulin (Gibco) at 37°C. The cell line was authenticated by Genetica DNA Laboratories using short tandem repeat DNA profiling and was found to be > 95% concordant. Cells were routinely tested for mycoplasma contamination and no contamination was detected throughout the duration of the study.

#### Collagen gels with cells

3D collagen gels were constructed as described in.^34^ Specifically, 200 *μ*l of Rat Tail Collagen I (RTCI; BD Biosciences, San Jose, CA, USA, 1.5 mg/ml final concentration) was incubated in a 20 mm glass well of a PELCO dish (#D35-20-1-N, *In Vitro* Scientific, Mountain View, CA, USA) at 37°C for half an hour until gel polymerization. 200 *μ*l of DOV13 cell suspension (50000 cell/ml) was applied on top of the polymerized collagen gel. Subsequently, the collagen construct with cells on top were introduced into an environmental chamber of a confocal microscope for high-resolution imaging.

### Method details

#### Confocal microscopy

DO13-GFP cells and collagen were observed with Nikon A1R-MP laser confocal microscope (Nikon Instruments, Inc., Melville, NY, USA) with ApolWD 30 x WI lambda-S DIC N2 water immersion objective lens. A series of high-resolution 100-*μ*m thick *z*-stacks of cells and collagen images was taken right after deposition of cells on the collagen, and subsequently, at 24 h and 48 h at least 5 different sites for each construct. An Argonne laser with 488-nm wavelength and helium-neon laser with 594-nm wavelength were used for fluorescence confocal and reflectance imaging of the labeled cells and unlabeled collagen matrix. The dimensions of an individual voxel of each z-stack were 0.2076 × 0.2076 × 0.6896 $\mu m^{3}$. During imaging, the samples were kept within the microscope environmental chamber to maintain 37*°*C and 5% ${\text{CO}}_{2}$.

#### Image analysis

Fiji software was used for the postprocessing of the acquired *z*-stack images and subsequent analysis. Standard Fiji plugins were utilized to evaluate the sizes and shape parameters of individual cells and cell clusters.

#### Numerical algorithm

Numerical simulations of the coupled non-dimensionalized system of Partial Differential Equations (PDEs) (1)-(5) are performed on a 2D-periodic domain of size L×L where L=20. The simulations use a finite differences method with a time and spatial steps of $\Delta t=5\times 10^{-3}$ and $\Delta x=10^{-2}$, respectively. Diffusion term in Equation 2 is approximated by the Euler-Maruyama scheme. PDEs (3) and (4) take the form $\partial_{t}v=Lv+f\left(v\right)$, where *L* is the second order derivative operator, and *f* is the remainder. These equations are discretized as $u^{n+1}-u^{n}=\Delta t(Lu^{n+1}+f\left(u^{n}\right)$) and the operator I−ΔtL is inverted using the Gauss-Seidel iterative scheme.

### Quantification and statistical analysis

#### Statistical analysis of experimental data

Statistical analysis of experimental data was performed using GraphPad 10 software. Statistical significance between groups of samples was determined using Mann-Whitney *U* test with a 95% confidence level. Results are presented as a median with 95% confidence level. $N_{c}=40$ cells of three replicates were analyzed for cellular size and shape quantification.

#### Cluster anisotropy

Two methods were used to determine the cluster elongation.

(i) by finding the direction of line l crossing the center of the cluster and oriented such that the sum of distances between the line l and cells from the cluster is minimal:
  $$\sum_{i=1}^{m_{c}}\text{dist}\left(r_{i},l\right)\to \min\text{.}$$
(ii) by finding the eigendirection of the covariance matrix
  $$\left[\begin{array}{cc} \sum_{i=1}^{m_{c}}{\left(x_{i}-x_{c}\right)}^{2} & \sum_{i=1}^{m_{c}}\left(y_{i}-y_{c}\right)\left(x_{i}-x_{c}\right)\\ \sum_{i=1}^{m_{c}}\left(x_{i}-x_{c}\right)\left(y_{i}-y_{c}\right) & \sum_{i=1}^{m_{c}}{\left(y_{i}-y_{c}\right)}^{2} \end{array}\right]$$
  corresponding to the largest eigenvalue of this matrix.

The direction of the minor axis is defined as the one perpendicular to the major axis. Then we define the length and width of the cluster as the minimal segment containing the projection of this cluster onto the major and minor axis, respectively.

### Additional resources

No additional resources were used in this study.
