# Supplementary material for: Durotaxis and extracellular matrix degradation promote the clustering of cancer cells
Source: iScience. 2025 Jan 24;28(3):111883. doi: 10.1016/j.isci.2025.111883 (PMC11914804; doi:10.1016/j.isci.2025.111883)
Supplement: Document S1. Figures S1–S3 [file mmc1.pdf]

iScience, Volume *28*

Supplemental information

Durotaxis and extracellular matrix degradation promote the clustering of cancer cells

Mykhailo Potomkin, Oleg Kim, Yuliya Klymenko, Mark Alber, and Igor S. Aranson

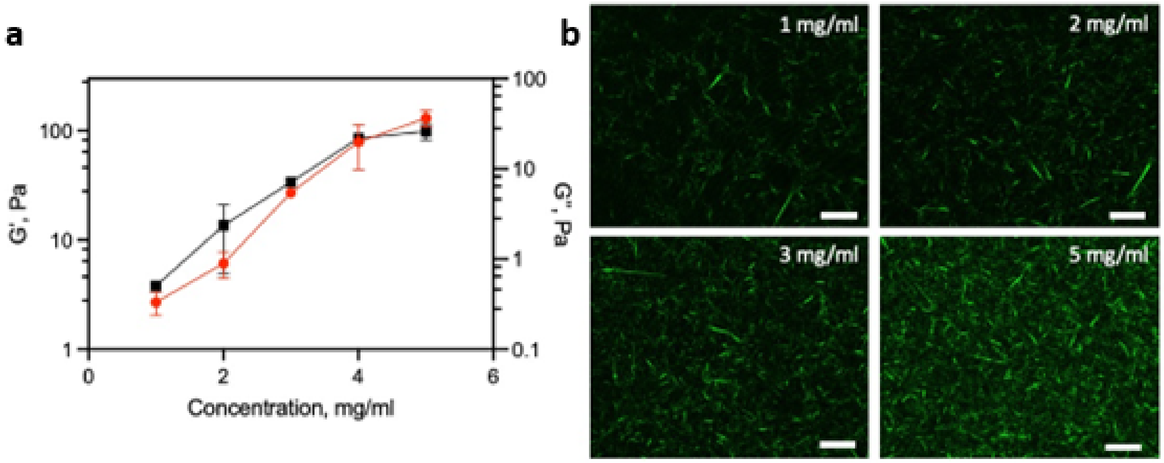

Figure S1: Viscoelasticity measurements and structure visualization of collagen matrices at different densities. Collagen gel shear viscoelasticity was measured using a rheometer (Discovery HR-30, TA instrument). To prepare collagen gels at different concentrations (1-5 mg/mL), type I collagen solution (stock concentration 12 mg/mL) was neutralized with 1 M NaOH and DMEM in appropriate ratios and the mixture was immediately loaded between the rheometer plates to initiate polymerization. The gel was polymerized in situ at 37°C for 20 min between parallel plates with a diameter of 20 mm and a gap distance of 500 mm. Following polymerization, oscillatory shear tests were conducted at a fixed frequency of 1 Hz and a strain amplitude of 0.8% which is within the linear viscoelastic range (0.1-1%). The shear and loss moduli (G', G") were determined from the resulting stress-strain data. Measurements were performed at room temperature (25°C) and analyzed using the rheometer's software (TRIOS). Each sample was measured in triplicate to ensure reproducibility, and results were expressed as mean ± standard error of the mean (SEM). Sub-figure **a**: Shear and loss moduli of collagen gels determined from shear oscillatory rheology. Sub-figure **b**: Second harmonic generation (SHG) microscopy imaging of the gels at different collagen concentrations. Scale bar, 10 μm.

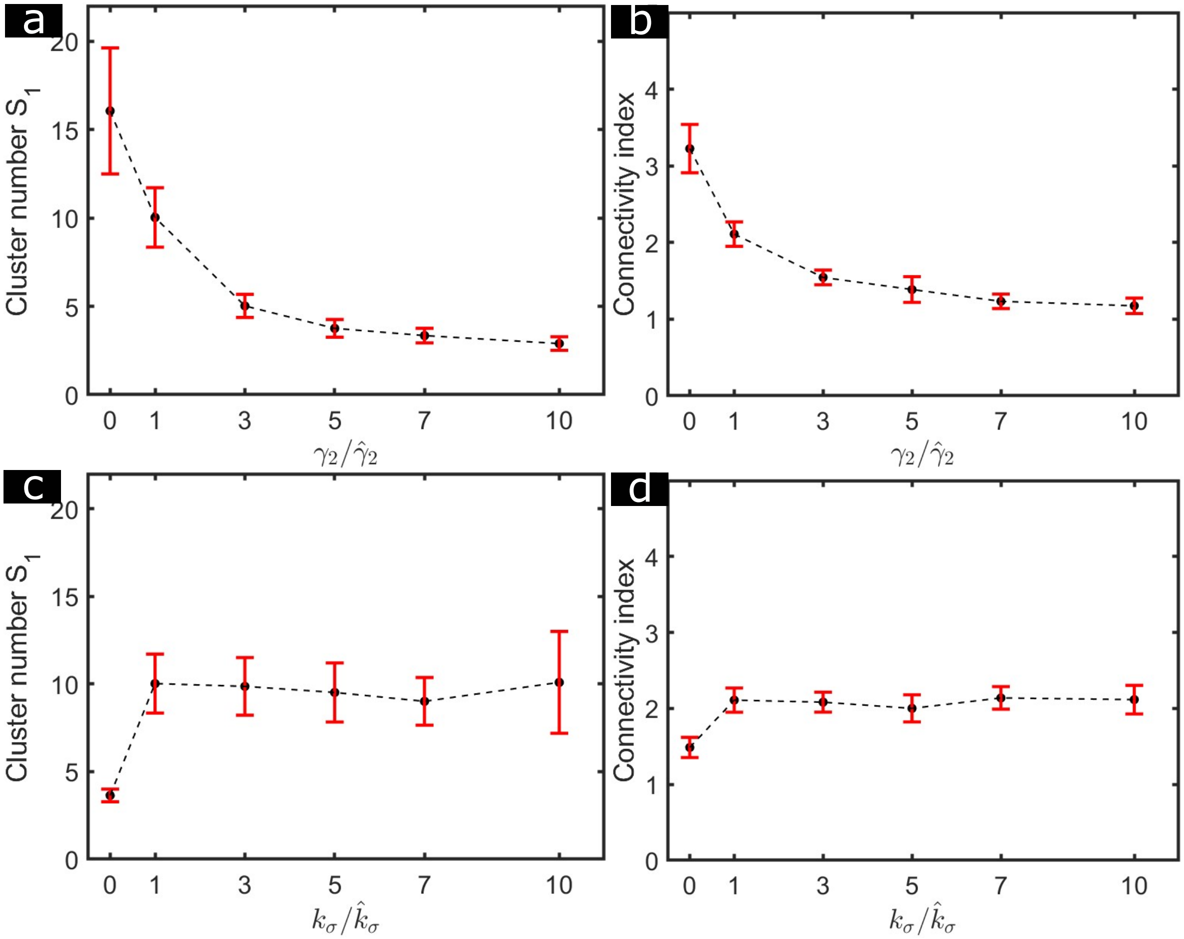

Figure S2: Results of numerical simulations for various values of degradation parameter γ_2 and durotaxis parameter k_σ. Here, $\hat{\gamma}_2$ and $\hat{k}_\sigma$ are values of parameters used in the main manuscript to study the case with both durotaxis and degradation. When varying one of these parameters, the other parameters are held at their typical values. For each choice of parameters we perform $R = 20$ independent simulations. Black points indicate the average value of either cluster number S_1 (sub-figures **a** & **c**)) or the connectivity index (sub-figures **b** & **d**)). Red bars correspond to the confidence interval for the level 95 %.

a *Nondimensionalization.* Introduce the following non-dimensional variables:

$$\hat{t} = \frac{V_{\rm prop} t}{d}, \quad \hat{\boldsymbol{x}} = \frac{\boldsymbol{x}}{d}, \quad \hat{\boldsymbol{r}} = \frac{\boldsymbol{r}}{d}, \quad \hat{\boldsymbol{u}} = \frac{\boldsymbol{u}}{d}, \quad \hat{C} = \frac{V_{\rm prop} C}{\nu_C d}.$$

b

$$\partial_{\hat{t}} \hat{\boldsymbol{r}}_i = \rho \boldsymbol{p}_i + \frac{\phi_0}{V_{\rm prop}} \sum_{j=1, j\neq i}^{N_c} \hat{\phi}(|\hat{\boldsymbol{r}}_j - \hat{\boldsymbol{r}}_i|) \frac{\hat{\boldsymbol{r}}_j - \hat{\boldsymbol{r}}_i}{|\hat{\boldsymbol{r}}_j - \hat{\boldsymbol{r}}_i|}, \quad i = 1, .., N_c.$$

$$\partial_{\hat{t}} \varphi_i = \frac{k_\sigma d^2}{V_{\rm prop}} (|\hat{\boldsymbol{u}}(\hat{\boldsymbol{x}}_{\rm right}, t)| - |\hat{\boldsymbol{u}}(\hat{\boldsymbol{x}}_{\rm left}, t)|) + \sqrt{\frac{2\hat{D}_\varphi d}{V_{\rm prop}}} \dot{\hat{W}}_i, \quad i = 1, .., N_c.$$

$$\partial_{\hat{t}} \hat{\boldsymbol{u}} = \frac{\hat{E}_0}{2\eta V_{\rm prop}} \nabla_{\hat{\boldsymbol{x}}} \cdot \left[(1 + \epsilon\rho)(\nabla_{\hat{\boldsymbol{x}}} \hat{\boldsymbol{u}} + (\nabla_{\hat{\boldsymbol{x}}} \hat{\boldsymbol{u}})^{\rm T})\right] + \frac{\zeta}{\eta V_{\rm prop}} \sum_{i=1}^{N_c} \hat{T}_i \left(|\hat{\boldsymbol{r}}_j - \hat{\boldsymbol{r}}_i|\right).$$

$$\partial_{\hat{t}} \hat{C} = \frac{D_C}{V_{\rm prop} d} \Delta_{\hat{\boldsymbol{x}}} \hat{C} + \sum_{i=1}^{N_c} \hat{w}(|\hat{\boldsymbol{x}} - \hat{\boldsymbol{r}}_i|) - \frac{\hat{\gamma}_1 d}{V_{\rm prop}} \hat{C} \rho - \frac{\beta d}{V_{\rm prop}} \hat{C}.$$

$$\partial_{\hat{t}} \rho = -\frac{\hat{\gamma}_2 \nu_C d^2}{V_{\rm prop}^2} \hat{C} \rho.$$

Figure S3: Computational model (1)-(5) in a non-dimensionalized form. Unknown functions are $\hat{\boldsymbol{r}}_1(\hat{t})$,...,$\hat{\boldsymbol{r}}_{N_c}(\hat{t})$, $\varphi_1(\hat{t})$,...,$\varphi_{N_c}(\hat{t})$, $\hat{\boldsymbol{u}}(\hat{\boldsymbol{x}}, \hat{t})$, $C(\hat{\boldsymbol{x}}, \hat{t})$, and $\rho(\hat{\boldsymbol{x}}, \hat{t})$. Constant parameters are $V_{\rm prop}$, ϕ_0, d, k_σ, $D_{\rm rot}$, η, ϵ, E_0, D_C, C_0, γ_1, β, ζ, γ_2. Functions $\hat{\phi}(z)$, $\hat{w}(z)$, $\hat{T}(z)$, are given by $\hat{\phi}(z) = -\hat{w}(z) = -e^{-z^2}$, $\hat{T}(z) = H(1-z)\left[\hat{w}(z) - \langle \hat{w} \rangle\right]$, where $H(s)$ is the Heaviside function and $\langle \tilde{w} \rangle$ is chosen so that a cell does not exert a net force on the substrate, $\int_{\mathbb{R}^2} \hat{T}(|\boldsymbol{r}|)\, \mathrm{d}\boldsymbol{r} = 0$. Locations of "sides", $\hat{\boldsymbol{x}}_{\rm right}$ and $\hat{\boldsymbol{x}}_{\rm left}$, of the ith cell are given by $\hat{\boldsymbol{x}}_{\rm right} = \hat{\boldsymbol{r}}_i + \hat{\ell}(\boldsymbol{p}_i \times \boldsymbol{e}_z)$ and $\hat{\boldsymbol{x}}_{\rm left} = \hat{\boldsymbol{r}}_i - \hat{\ell}(\boldsymbol{p}_i \times \boldsymbol{e}_z)$, $\boldsymbol{e}_z = (0, 0, 1)^{\rm T}$. Sub-figure **a** introduces non-dimensional variables. Sub-figure **b** presents the non-dimensional model.
